# Fcγ-Receptor-Independent Controlled Activation of CD40 Canonical Signaling by Novel Therapeutic Antibodies for Cancer Therapy

**DOI:** 10.3390/antib13020031

**Published:** 2024-04-18

**Authors:** Karsten Beckmann, Carmen Reitinger, Xianglei Yan, Anna Carle, Eva Blümle, Nicole Jurkschat, Claudia Paulmann, Sandra Prassl, Linda V. Kazandjian, Karin Loré, Falk Nimmerjahn, Stephan Fischer

**Affiliations:** 1 Biontech SE, Forstenrieder Str. 8-14, 82061 Neuried, Germany; 2 Division of Genetics, Department of Biology, Friedrich-Alexander-University Erlangen-Nürnberg, 91058 Erlangen, Germany; 3 Division of Immunology and Allergy, Department of Medicine Solna, Karolinska Institutet, Karolinska University Hospital, Visionsgatan 4, BioClinicum J7:30, 171 64, Stockholm, Sweden; 4 Center of Molecular Medicine, 171 76, Stockholm, Sweden; 5 FAU Profile Centre Immunomedicine, 91054 Erlangen, Germany; 6 Icanomab, Tassilostr. 2, 82398 Polling, Germany

**Keywords:** CD40, antibody, dendritic cell, immunoglobulin, immune cell activation

## Abstract

The activation of CD40-mediated signaling in antigen-presenting cells is a promising therapeutic strategy to promote immune responses against tumors. Most agonistic anti-CD40 antibodies currently in development require the Fcγ-receptor (FcγR)-mediated crosslinking of CD40 molecules for a meaningful activation of CD40 signaling but have limitations due to dose-limiting toxicities. Here we describe the identification of CD40 antibodies which strongly stimulate antigen-presenting cells in an entirely FcγR-independent manner. These Fc-silenced anti-CD40 antibodies induce an efficient upregulation of costimulatory receptors and cytokine release by dendritic cells. Finally, the most active identified anti-CD40 antibody shows activity in humanized mice. More importantly, there are no signs of obvious toxicities. These studies thus demonstrate the potent activation of antigen-presenting cells with anti-CD40 antibodies lacking FcγR-binding activity and open the possibility for an efficacious and safe combination therapy for cancer patients.

## 1. Introduction

Over the past decades, a number of therapeutic strategies have been developed aiming to support or enhance tumor specific immunity in cancer patients. Checkpoint inhibitors (CPIs) like PD1/PDL1-and CTLA-4-targeting antibodies have proven to be effective in several cancer types, yet a large fraction of patients whose tumor microenvironment is often classified as “cold” or non-inflamed do not respond to CPI therapy [1]. In addition to testing combinations of different CPIs, recent approaches to tackling resistance to CPI therapy include CAR-T cell therapies or vaccinations with personalized neo-antigens. However, such therapies are expensive and require a large infrastructure, and the therapeutic effects have been mixed. Alternatively, agonistic antibodies, triggering critical co-stimulatory pathways for cytotoxic and T-helper cell activation and differentiation, have demonstrated very promising activity in cancer immunotherapy [2]. The CD40-CD40L-signaling axis is a powerful, well-studied mechanism that is key for the generation of new, antigen-specific T and B cell responses. Professional antigen-presenting cells (APC), such as dendritic cells (DC), express CD40 and become stimulated by CD40L on T-helper cells in the course of antigen recognition. As a consequence, CD40 signaling licenses APCs for the stimulation of antigen-specific CD8 T-cell responses by the upregulation of costimulatory surface receptors and the release of T-cell-activating cytokines [3]. The therapeutic triggering of canonical CD40 signaling is particularly attractive, since it provides an opportunity to elicit novel patient- and tumor-specific immune responses using a conventional biological, e.g., an agonistic antibody [4,5]. Experiments in mice have provided extensive support for this therapeutic strategy. In early studies, FGK-45, an agonistic anti-murine-CD40 antibody, was shown to stimulate cytotoxic T-cell responses and to provide protection against tumor re-challenge [6]. A local intra-tumoral injection of FGK-45 has further demonstrated the systemic licensing of tumor-reactive cytotoxic T-cells resulting in the eradication of distant tumor nodules [7]. 

The use of F(ab)_2_ antibody fragments and Fcγ-receptor-(FcγR) knock-out mice revealed that the activity of FGK-45 and other CD40 antibodies is largely mediated by the FcγR-directed crosslinking of CD40 molecules. In particular, FcγRIIb has been identified as a potent enhancer of anti-CD40 antibody-mediated activities [8,9,10,11,12,13]. For certain IgG2 Fc-variants an alternative CD40 crosslinking mechanism depending on the antibody hinge conformation has been demonstrated to confer enhanced agonistic antibody activity [14,15,16]. In general, a higher order FcγR-mediated crosslinking of agonistic antibodies bound to their target molecule has been established as a major mode of action for most TNFR family-targeting agonistic antibodies [2,17,18]. However, it was also recognized in many studies that treatment with CD40 antibodies is associated with severe side effects. For example, FGK-45, depending on the site and schedule of administration, elicited shock syndrome, liver damage and thromboembolic events and caused lethality [7,19,20]. There is evidence indicating that at least for certain CD40 antibodies FcγR binding may contribute to these adverse events [8,21]. Interestingly, the association between FcγR binding and adverse events is not restricted to CD40 antibodies but has also been observed in antibodies targeting other TNFR family members or CD40L [9,22,23,24]. 

Nevertheless, therapeutic CD40 antibodies entered clinical development more than ten years ago. Their pharmacological profile in humans is reminiscent of what has been observed in animal models. The best-studied antibody, CP-870,893, showed clinical activity but also severe side effects. Dose-limiting toxicities were thromboembolic events and liver enzyme elevations resulting in a maximum tolerated dose in patients of 0.2 mg/kg [25,26,27]. Fc-modified second generation CD40-specific antibodies with increased binding specificity for FcγRIIb have been generated to achieve a better therapeutic window [2,18]. In addition, intra-tumoral application has been proposed for clinical use to circumvent systemic side-effects [8,12,21]. More recently, epitope specificity and affinity, as well as IgG isotype, were shown to impact the activity of human CD40-specific antibodies, creating a complex scenario in which antagonistic antibodies may be turned into agonistic antibody species [28,29,30]. 

Based on this preclinical and clinical experience, we reasoned that developing novel agonistic CD40-specific antibodies with an entirely FcγR-independent mode action may be a strategy to create candidate antibodies with a less complex pharmacokinetic and reduced systemic toxicity. However, this mode of action requires that canonical CD40 signaling is efficiently triggered by the crosslinking of CD40 but without the necessity for higher-level crosslinking via the Fc-part. The generation of such antibodies in immunized animals, however, is a very rare event. In this study we describe the generation of novel human CD40-targeting antibodies and their characterization in vitro and in vivo. We used the immunization of wildtype rabbits combined with direct B-cell cloning, high-throughput functional screening and humanization to isolate such antibodies. Indeed, this approach allowed for the isolation of large numbers of diverse agonistic human anti-CD40 antibody candidates, from which several antibody clones with a high agonistic activity and low systemic toxicity could be isolated. 

## 2. Material and Methods

### 2.1. Antibodies and Reagents

CD86-VioBright-515, CD80-PE and HLA-DR-VioBlue antibodies and isotype controls were from Miltenyi Biotec (Bergisch Gladbach, Germany). Recombinant CD40L protein, containing a mouse-IgG2a Fc-tag, was from AB Biosciences (#P7005F; Concord, MA, USA). HIS-tagged CD40 recombinant protein was from Acro Biosystems (Basel, Switzerland). Human-IgG2, IgG1, IgG1-V11 and IgG1-LALA antibody variants of CP870,893 contain the variable region sequences of the clone 21.4.1 described in patent US 7,338,660. APX is a hIgG1-S267E anti-CD40 antibody containing the variable region sequences of the antibody APX005 described in patent US 2014/0120103. MAB 273, 271, 276 and 278 antibodies were identified and cloned from human-CD40-immunized New Zealand white rabbit B-cells. The humanization of the rabbit antibodies was performed by Fusion Antibodies plc (Belfast, Northern Ireland, UK) by using proprietary technology. In short optimal human frameworks for the rabbit CDR antibody regions are identified by an in silico approach followed by the recombinant production and testing of humanized antibody candidates. Variable regions were fused to hIgG1 Fc sequences containing L234A and L235A mutations [31]. Antibodies were produced at MAB Discovery GmbH using the FreeStyle™ 293 expression system from Thermo Fisher (Waltham, MA, USA) (catalogue number: K900001). Transfected HEK-293 FreeStyle™ cells were incubated for 10–11 days at 37 °C. The harvesting of cell supernatants was achieved with a two-step centrifugation procedure, followed by a filtration step. Antibody purification from cell supernatants was carried out in two steps using the ÄKTA Avant purification system (GE Healthcare, Chicago, IL, USA). The antibodies were purified by affinity chromatography using a Protein A resin (MabSelect SuRe, GE Healthcare), followed by a preparative size exclusion chromatography (SEC). All preparations were endotoxin-low (<0.05 EU/mg) as determined by the EndoZyme^®^ assay (Hyglos GmbH, Bernried, Germany). For specific antibodies an analytical size exclusion chromatography was performed to determine the aggregation state and the size in solution. Briefly, a size standard was established by using bovine blood IgG (150 kDa), bovine thyroglobin (67 kDA), ovalbumin (44.4 kDa) and ribonuclease type I-A (13.7 kDa) before running different antibody preparations on the column (MabPac SEC-1) at 0.25 mL/min under two buffer conditions (mobile phase 1: 100 mM phosphate, 200 mM arginine, pH6,4; mobile phase 2: 100 mM phosphate, 300 mM NaCl, pH6,4).

### 2.2. HEK-Blue-CD40L^TM^ Cell-Based Gene Reporter Assay

The agonistic activity of anti-CD40 monoclonal antibodies was tested by stimulating HEK-Blue-CD40L™ (Invivogen, San Diego, CA, USA, catalogue code: hkb-cd40) cells, which harbor an NF-κB-inducible Secreted Embryonic Alkaline Phosphatase (SEAP) gene construct. Briefly, 25,000 HEK-Blue-CD40L™ cells/well in 20 μL DMEM containing 10% FBS were seeded in a cell-culture 384-well plate and cultured overnight. Humanized anti-CD40 hIgG1-LALA antibodies were added in a volume of 5 μL medium to final concentrations ranging from 10 to 0.08 μg/mL. After 6 h of incubation at 37 °C and 5% CO_2_, 5 μL of the medium supernatant of each well was transferred to a white clear-bottom 384-well plate containing 20 μL of 2× QUANTI-Blue™ reagent (InvivoGen, San Diego, CA, USA). After incubation at 37 °C and 5% CO_2_ for one hour, optical density at a wavelength of 655 nM was measured reflecting the NF-κB-dependent activation of phosphatase secretion. 

### 2.3. Induction of Costimulatory Receptors and Cytokine Release in Dendritic Cells

Buffy coats from different anonymous donors were provided by the Bavarian Red Cross. Monocytes were isolated from buffy coat-derived PBMCs by CD14 MACS (Miltenyi, Bergisch Gladbach, Germany) according to the supplier’s protocol and differentiated to immature DCs (iDCs) for 5 days in the presence of 50 ng/mL hGM-CSF and 10 ng/mL hIL-4. Anti-CD40 or isotype control antibodies were added to iDCs in a 96-well plate (10^6^ cells/mL) at concentrations ranging from 10,000 to 5 ng/mL. For analysis of costimulatory receptors, DCs were harvested 48 h after the addition of antibodies and stained using fluorophore-labeled antibodies against HLA-DR, CD86 and CD80. Analysis was performed by flow cytometry on a BD FACSVerse device (Becton Dickinson GmbH, Heidelberg, Germany). Cytokine concentrations in the supernatants of DC cultures were measured using a BD human inflammatory cytometric bead array kit (BD #551811) according to the manufacturer’s instructions. IL-12p40 cytokine release was quantified using the Duo Set ELISA kit from R&D Systems (Wiesbaden, Germany) according to the manufacturer’s instructions. Absorbance at 450 and 620 nm wavelength was measured using a Tecan (Crailsheim, Germany) M1000 microplate reader. Fitting curves were calculated using GraphPad Prism software version 10.1 (Boston, MA, USA).

### 2.4. Induction of Costimulatory Receptor Expression on B-cells

PBMCs from three different anonymous donors were isolated from human buffy coats by Ficoll density gradient centrifugation, and untouched B-cells were purified by negative magnetic enrichment using a B-cell isolation kit II (Miltenyi Biotec) according to the manufacturer’s instructions. 2 × 10^5^ B-cells in 100 μL RPMI-1640 + 10% Human AB Serum were stimulated with CD40 antibody concentrations ranging from 500 to 0.2 ng/mL for 48 h. Stimulated B-cells were harvested, stained using fluorophore-labeled antibodies against CD86 and CD80 and analyzed by flow cytometry on a BD FACSVerse device. Fitting curves were calculated using GraphPad Prism.

### 2.5. Competition of CD40 Antibodies with CD40L Binding to Cell-Expressed CD40

HEK-Blue™-CD40L cells (Invitrogen, catalogue code: hkb-cd40) were pre-incubated with antibodies at their EC90 binding concentration for 30 min at 4 °C. Recombinant CD40L protein was added at concentrations ranging from 10,000 to 9.8 ng/mL, and cells were incubated for 60 min at 4 °C. CD40L bound to cell-expressed CD40 was detected using secondary DyLight 405-conjugated anti-mouse IgG (Jackson Laboratories, Bar Harbor, ME, USA), while the anti-CD40 antibodies were detected using an Alexa Fluor 488-conjugated goat anti-human-F(ab)_2_ (Jackson ImmunoResearch, Cambridgeshire, UK). Cells were analyzed using a FACSVerse instrument (Becton Dickinson GmbH, Heidelberg, Germany).

### 2.6. Competition of Anti-CD40 Antibodies for Binding to Human CD40

Antibodies were coated to 384-well Maxisorp plates at a concentration of 625 ng/mL in PBS for 60 min followed by a blocking step with PBS, 2% BSA, 0.05% Tween for 70 min. All antibodies were incubated separately at a concentration of 10 μg/mL in tubes together with 330 ng/mL HIS-tagged CD40 recombinant protein and 4 μg/mL peroxidase-coupled anti-HIS detection antibody (Sigma-Aldrich, St. Louis, MO, USA) for 60 min in ELISA buffer (PBS, 0.5% BSA, 0.05% Tween). The plate was washed three times with PBS containing 0.1% Tween before the antibody/HIS-CD40/anti-HIS-peroxidase mixes were added to the wells of the plate. The plate was incubated for 60 min. Wells were washed six times with PBS and 0.1% Tween, and 15 μL/well TMB substrate solution (Invitrogen, Waltham, MA, USA) was added. The reaction was stopped with 15 μL/well Stop solution (1M HCl), and absorbance at 450 and 620 nm wavelength was measured using a Tecan M1000 microplate reader.

### 2.7. Stem-Cell-Humanized Mouse Model

Animal work was performed in accordance with the guidelines of the National Institutes of Health and the legal requirements in Germany and approved by the local review board (#55.2-2532-2-817). Human stem cells were purified from anonymous human umbilical cord blood with the written consent of cord blood donors and according to the ethical guidelines of the University of Erlangen (approval #4414). Nod/Scid/IL2rg^−/−^/FcRγ^−/−^ (female and male mice) were generated by crossing the individual mouse strains, followed by backcrossing to the Nod/Scid background. Mice were irradiated sub-lethally with 1.4 Gy within the first 24 h after birth, followed by engraftment with 20,000–50,000 human hematopoietic stem cells isolated from umbilical cord blood (hCD34 + MACS). When the mice were aged 10–12 weeks the efficiency of humanization in the peripheral blood was verified via FACS analysis. Successfully humanized mice (≥5% hCD45 + cells) were injected i.v. with the indicated amounts of anti-CD40 antibodies or isotype control antibodies in 100 μL 1xPBS. Body weight and temperature were measured before treatment and at the indicated time points after antibody injection. 

### 2.8. Analysis of Human Leucocytes in Murine Peripheral Blood

To determine relative (or absolute) changes in white blood cell composition in the blood, FACS analysis was performed. Peripheral blood (100μL) was collected by retro-orbital or venal puncture. Erythrocytes were lysed by adding 900 μL of ddH_2_O for 20 s. After the addition of 100 μL of 10x PBS the reaction was stopped, and the probes were centrifuged at 600× *g* for 5 min at room temperature. The cell pellet was re-suspended in Fc-Block (0.5 μg/well, 2.4G2) and incubated for 15 min at 4 °C. After one centrifugation step (1400 rpm, 5 min, 4 °C) the cells were stained with fluorochrome-conjugated antibodies for FACS analysis (all antibodies from Biolegend, San Diego, CA, USA) for 15 min at 4 °C. For live/dead cell exclusion DAPI was added at a dilution of 1:5000. After centrifugation (1400 rpm, 5 min, 4 °C) cells were re-suspended in 100 μL FACS buffer and analyzed on a FACS Canto II (Becton Dickinson GmbH, Heidelberg, Germany). 

### 2.9. Serum Cytokine Analysis

Human cytokine levels in humanized mice were detected by using a LEGENDplex^TM^ Multi-Analyte Flow Assay Kit as instructed by the manufacturer. In brief, peripheral blood of humanized mice was obtained as described above and centrifuged at 10,000× *g* for 5 min, followed by the storage of collected sera at −80 °C. Samples were diluted 1:2 with assay buffer and 12.5 μL of assay buffer, sample or standard, mixed beads and detection antibody. The mixture was incubated at RT for 2 h on a shaker (1000 rpm). 12.5 μL of SA-PE/well was added, and samples were shaken at 1000 rpm at RT for 30 min. After one washing step beads were re-suspended in 200 μL wash buffer and analyzed on a FACS Canto II. 

### 2.10. Immunohistochemistry of Liver and Kidney

Liver and kidneys were frozen at −80 °C in OCT, cut into 5 μM sections and fixed with acetone for 2.5 min. Sections were incubated in blocking solution (5% goat serum in PBS) for 1 h at RT before fluorochrome-conjugated antibodies were added in 5% goat serum in PBS and incubated for 30 min at RT in the dark. Slides were rinsed three times with 1xPBS, mounted with a drop of mounting medium and dried for 30 min at RT in the dark. Stained sections of liver and kidney were analyzed on an Axiovert 200 M (Zeiss, Jena, Germany).

### 2.11. AST/ALT Measurement

Concentrations of aspartate aminotransferase (AST) and alanine aminotransferase (ALT) in serum samples were determined using an Alanine Aminotransferase (ALT) Activity Colorimetric Assay Kit and an Aspartate Aminotransferase (AST) Activity Colorimetric Assay Kit from Biovision:Abcam (Cambridge, UK) following the manufacturer’s instructions.

### 2.12. Surface Plasmon Resonance Measurements

SPR measurements were performed by Biaffin GmbH (Kassel, Germany). Briefly, antibodies were reversibly immobilized to a CM5 sensor chip surface via an anti-human Fc antibody. Serial threefold dilutions of HIS-tagged, human CD40 recombinant protein were analyzed in duplicate to kinetically characterize interactions. The kinetics of the interaction of immobilized antibodies with soluble CD40 were analyzed on a Biacore T200 SPR (Cytiva, Marlborough, MA, USA) instrument. Kinetic data were determined using a Langmuir 1:1 binding model.

## 3. Results

### 3.1. Identification of Novel CD40-Specific Agonistic Antibodies

**Figure 1:**
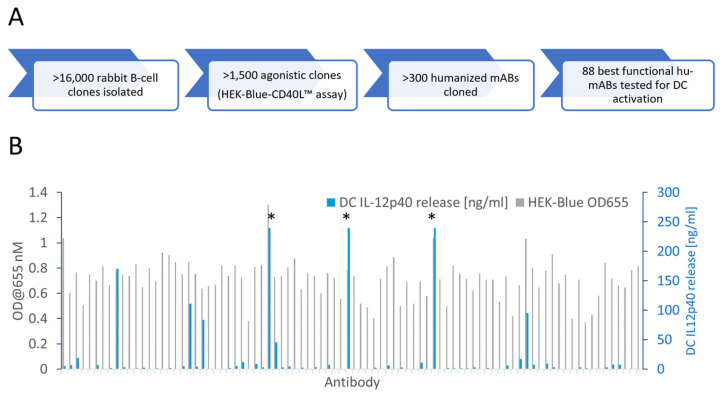
Identification of agonistic anti-CD40 antibodies not requiring Fc-effector function. (**A**) Flow diagram of the anti-CD40 antibody discovery campaign. Wildtype rabbits were immunized with recombinant CD40 protein and monoclonal B-cells were FACS-sorted and cultured. Antibodies secreted into the B-cell supernatant medium were screened for the induction of NFκB signaling in a HEK-Blue-CD40L™ cell-based gene reporter assay. Antibody gene sequences were retrieved from B-cells, humanized and cloned as human IgG1-LALA antibodies in HEK-293 FreeStyle™ cells. (**B**) Eighty-eight humanized anti-CD40 hIgG1-LALA antibodies were tested on CD40 expressing HEK 293 cells in the HEK-Blue-CD40L™ cell-based gene reporter assay (grey bars; OD@655 = secreted embryonic alkaline phosphatase activity) and for the induction of IL-12p40 cytokine release by in vitro differentiated dendritic cells (blue bars; measured by ELISA). Asterisks (*) indicate values exceeding the linear range of the ELISA.

Wildtype rabbits are a unique source of therapeutic antibodies because of their exceptional mechanisms for diversifying antibody gene sequences. Common VDJ recombination and somatic hypermutation mechanisms are enforced by V-gene conversion, which proceeds to diversify the antibody repertoire even during the course of an immunization, thus leading to diverse and high-affinity antibodies [32,33]. We immunized rabbits with recombinant human CD40, isolated monoclonal B-cell clones by flow cytometry (FACS) and functionally tested >16,000 antibodies secreted into the supernatants of cultured monoclonal B-cells (Figure 1A). A high-throughput NFκB gene reporter screen identified 1500 agonistic anti-CD40 antibody candidates. VL and VH genes of more than 300 antibody candidates were sequenced from B-cell lysates. We generated one humanized V-region sequence for each rabbit V-region sequence applying a structural modelling-assisted CDR grafting process developed by Fusion Antibodies PLC. Humanized V-regions were cloned into a human IgG1 backbone containing the L234A and L235A (LALA) mutation to prevent FcγR binding [31]. Out of a total of 303 candidate antibodies 198 remained functional after humanization and recombinant production as hIgG1-LALA antibodies (Figure S1). Figure 1B shows the activity of 88 selected humanized anti-CD40 hIgG1-LALA antibodies in the HEK-Blue™ NFκB gene reporter assay, demonstrating that all 88 antibodies confer agonistic signaling upon binding to CD40. 

### 3.2. FcγR-Independent Activation of Innate Immune Responses

For a more in-depth characterization of agonistic activity, we established a primary cell-based test system. Primary as well as in vitro differentiated monocyte-derived DCs (moDC) in mice and humans are known to express FcγRs and trigger T cell activation [34,35,36]. Thus, the stimulation of moDC maturation is a suitable assay to test whether antibodies induce the activation of CD40 signaling dependent on or independent of FcγR-mediated crosslinking. As the production of IL-12p40 by DCs is a hallmark of DC maturation, we used IL-12p40 release as a measure of CD40-antibody-dependent moDC activation [37]. As shown in Figure 1B, a small fraction of the IgG1-LALA antibodies induced high levels of IL-12p40 release by DCs, while the majority of antibodies showed low or absent activity in the hIgG1-LALA format.

**Figure 2:**
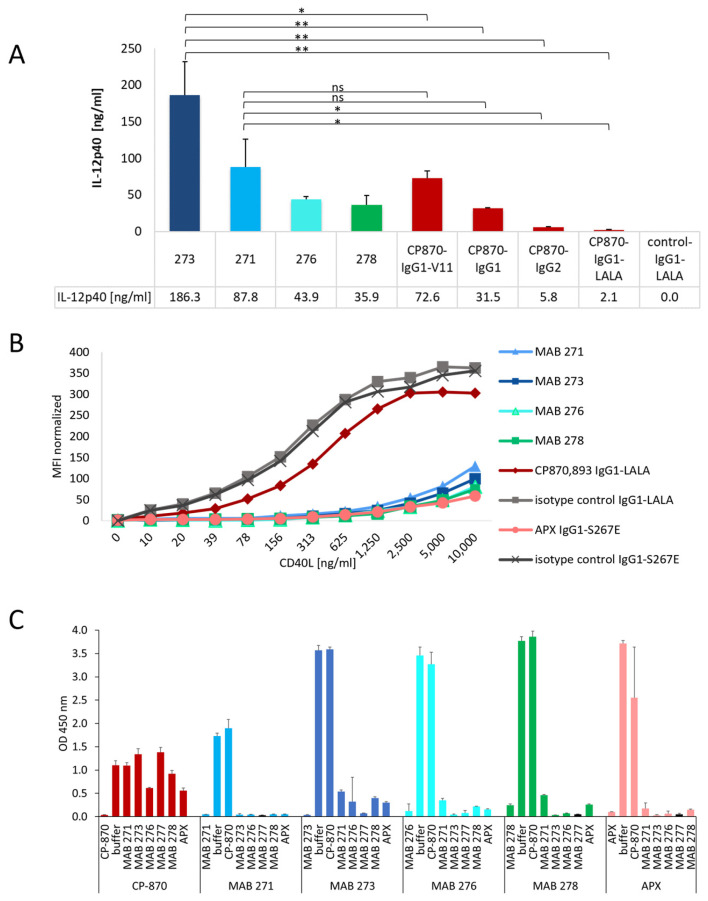
Differentiation of novel Fcγ-receptor-independent anti-CD40 antibodies from other clinical antibodies. (**A**) The activity of the four most active CD40 hIgG1-LALA antibodies, MAB 271, 273, 276 and 278, were tested for the activation of in vitro differentiated DCs in direct comparison to different Fc-variants of CP-870,893. IL-12p40, released by DCs, was measured by ELISA. The statistical significance for difference between MAB 273, MAB 271 and the Fc-variants of CP-870,893 was determined by an unpaired *t*-test. * *p* < 0.05, ** *p* < 0.01, ns = not significant. (**B**) CD40 antibodies were incubated with CD40-expressing HEK-Blue-CD40L™ cells, and interference with CD40L binding to CD40 was tested by the addition of increasing concentrations of recombinant CD40L-mIgG2a-Fc-fusion protein. The binding of CD40L was detected by flow cytometry using a fluorescently labeled anti-mouse IgG antibody. (**C**) The competition of anti-CD40 antibodies for binding to human CD40 was tested in a sandwich ELISA. Each antibody was tested both as a coating (vertical label) and as a detection antibody (horizontal label) in combination with any other anti-CD40 antibody. Error bars in (**A**,**C**) indicate the standard deviation (mean) of triplicates.

We next compared the capacity of the identified novel antibody LALA variants to trigger IL12p40 release by moDCs with Fc-variants of the clinical CD40-specific antibody CP-870,893, which contains a human IgG2 Fc-portion. In addition, we also generated hIgG1, hIgG1-V11 and hIgG1-LALA variants, which have a different capability to interact with FcγRs. Notably, the hIgG1-V11 variant increases the affinity for binding to the inhibitory FcγRIIb by more than 90-fold [8,38]. As shown in Figure 2A, the CP-870,893-hIgG1-V11 variant indeed showed the highest activity, followed by the hIgG1 and hIgG2 switch variants, consistent with previous studies [8,12]. As expected, the hIgG1-LALA version was functionally inactive, demonstrating the impact of FcγR binding on CP-870,893 activity in this assay system. Notably, the four tested novel CD40-specific hIgG1-LALA antibodies 271, 273, 276 and 278 induced either a comparable or an up to six-fold enhanced level of IL-12p40 release by DCs compared to the clinical CP-870,893-hIgG2 variant (Figure 2A). To test if the four antibodies harbor similar paratopes, we analyzed the antibody CDR-sequences to determine the degree of amino acid sequence divergence. As shown in Table S1, HC and LC CDRs differ by 20–58%, indicating that these antibodies are not related to each other at the sequence level. In summary, these results provide convincing evidence that we were able to identify a diverse set of CD40-specific antibodies with the ability to induce a strong CD40-dependent activation of antigen-presenting cells independently of FcγR-mediated crosslinking.

### 3.3. Impact of CD40-Specific Antibodies on CD40L Binding to CD40

Next, we investigated whether the four CD40-specific agonistic antibodies compete with CD40 ligand binding to CD40. As comparators we used CP-870,893, which is known not to bind the CD40L-binding region, and APX, a clinical-stage hIgG1 antibody with an S267E mutation in the Fc-part to increase FcγRIIb binding, which is known to compete with CD40L binding [29,39,40]. As shown in Figure 2B, all four identified CD40-specific antibodies and APX blocked the binding of increasing concentrations of mouse Fc-tagged CD40L to CD40-expressing HEK-Blue™ cells. As expected, isotype control and CP-870,893 antibodies did not interfere with CD40L binding, confirming the validity of the experimental system. We further tested the binding of the different anti-CD40 antibodies to HEK-Blue™ cells in the presence of CD40L. As shown in Figure S2, however, bound CD40L did not significantly affect anti-CD40 antibody binding to these cells, indicating that these antibodies cannot displace pre-bound CD40L. Next, we investigated the mutual competition of all antibodies for CD40-binding by using a sandwich ELISA setup where each antibody was tested either as a coating or as a detection antibody. In line with the results presented in Figure 2B, all CD40L-competing antibodies also competed with each other for binding to CD40, while none of these antibodies competed with the binding of CP-870,893 to CD40 (Figure 2C). 

### 3.4. Agonistic Antibody-Mediated Activation of CD40-Dependent Effector Pathways in Dendritic Cells 

**Figure 3:**
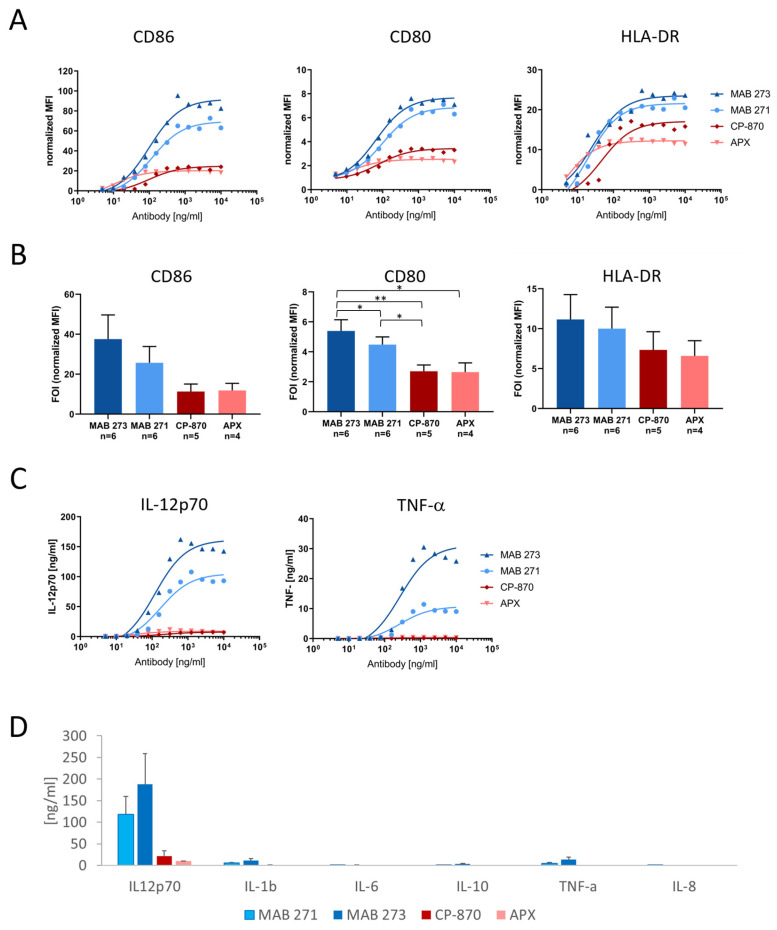
Induction of costimulatory receptors and cytokine release by Fc-silenced anti-CD40 antibodies in dendritic cells. Human PBMC-derived moDCs were treated for 2 days with anti-CD40 hIgG1-LALA antibodies MAB 271 and MAB 273, CP-870,893 hIgG2, APX hIgG1-S276E or isotype control antibodies. The expression of CD80, CD86 and HLA-DR was measured by flow cytometry (**A**,**B**), and cytokine concentrations in culture supernatants were determined by a cytometric bead array (**C**,**D**). MFIs were normalized to isotype control antibody treatments. In (**A**,**C**) a dose response analysis with DCs of an exemplary donor is shown; (**B**,**D**) summarize the mean of indicated donors treated with saturating concentrations of 1.25 or 2 μg/mL. Error bars (**B**,**D**) show SEM. Significant differences according to paired *t*-test are indicated in the bar diagrams. * *p* < 0.05, ** *p* < 0.01.

A hallmark of DC maturation is the upregulation of costimulatory receptors, such as CD80 and CD86, as well as MHC class II (MHCII) molecules. Thus, we investigated the capacity of the two most active anti-CD40 antibodies, MAB 271 and MAB 273, to upregulate CD80 and CD86 and the MHCII molecule HLA-DR on moDCs by flow cytometry. Neither antibody preparation contained aggregates, and both showed the expected size in solution as demonstrated by analytical size exclusion chromatography (Figure S3). As comparator antibodies we included CP-870,893-IgG2 and APX-hIgG1-S267E to cover both CD40L-competing and -non-competing FcγR-dependent anti-CD40 antibodies. As shown in Figure 3A, all antibodies induced co-receptor expression in a dose-dependent manner. Interestingly, MAB 273 and 271 treatment induced the highest expression levels of CD80 (Figure 3A,B). 

Next, we characterized the induction of the inflammatory cytokine secretion of anti-CD40-treated moDCs (Figure 3C,D). Mature IL-12p70, which is driving Th1 differentiation, is the cytokine most significantly induced by MAB 273 and 271, while the Th2 cytokine IL-10 is secreted at low levels (Figure 3C,D). Compared with MAB 273, CP-870,893 and APX induced a nine- or 18-fold lower IL12p70 cytokine release, respectively. The general response and absolute cytokine levels between different donors differed strongly, which explains why some of the differences between antibody treatments do not reach statistical significance even though the relative differences for each donor and experiment were consistent (as exemplified in Figure 3C). Furthermore, TNF-α and IL-1β, both known to stimulate or to be produced by DCs undergoing maturation, were produced at lower levels upon MAB 273 or MAB 271 treatment. In contrast, both of these cytokines were barely detectable upon moDC treatment with CP-870,893 or APX (Figure 3C,D). In summary, both MAB 273 and MAB 271 were efficiently able to induce moDC activation and trigger the secretion of a Th1-like cytokine profile.

Apart from binding to a relevant region on the CD40 receptor, the agonistic strength of antibodies may also be defined by its affinity. We measured the binding kinetics of the identified anti-CD40 and reference antibodies using SPR (Table S2). While MAB 271, CP-870,893 and APX bind to human CD40 with affinities ranging from 8.9 to 15.7 nM, MAB 273, which appears to provide the most potent agonistic effect, binds with a higher affinity of 1.2 nM. 

### 3.5. Agonistic Antibody-Mediated Activation of CD40-Dependent Effector Pathways in B Cells

**Figure 4:**
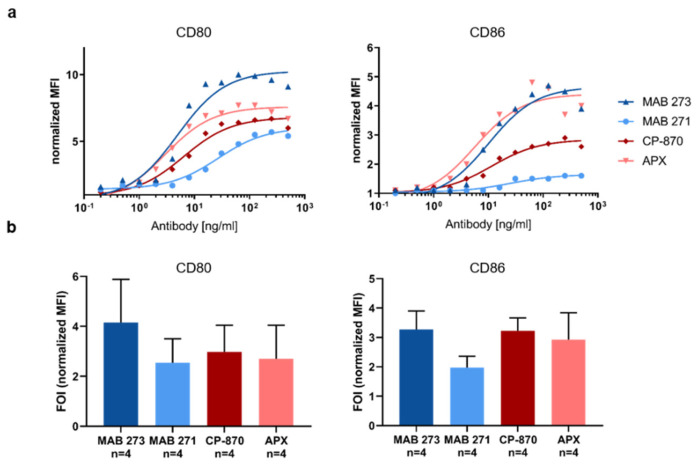
Fc-silenced anti-CD40 antibodies induce costimulatory receptors on human B-cells. B-cells were isolated from human PBMCs and incubated with anti-CD40 hIgG1-LALA antibodies MAB 271 and MAB 273, CP-870,893-hIgG2, APX hIgG1-S276E or isotype control antibodies. The expression of CD80 and CD86 was measured by flow cytometry. MFIs were normalized to isotype control antibody treatments. In (**a**), a dose response analysis with B-cells of an exemplary donor is shown; (**b**) summarizes the mean of four donors treated with saturating concentrations of 312 μg/mL (1 donor) or 500 μg/mL (3 donors). Error bars indicate SEM from four donors; the respective differences between anti-CD40 antibody treatments are not statistically significant according to paired *t*-test.

Besides DCs, CD40 is also expressed on B-cells, which respond to CD40 activation with an upregulation of CD80 and CD86 to increase antigen-presenting capabilities [41,42]. Therefore, we next determined the capacity of MAB 273, MAB 271, CP-870 and APX to induce the upregulation of these costimulatory receptors on B-cells isolated from human PBMCs. Compared with moDC activation, we observed a less pronounced upregulation of CD80 and CD86 induced by the antibodies (Figure 4). As observed in moDCs, all antibodies induced CD80 and CD86 expression in a dose-dependent manner (Figure 4a). However, as observed for the moDC costimulatory receptor and cytokine release analysis, the upregulation of both receptors on B-cells varied significantly from donor to donor. Overall, the ability of MAB 273, CP-870,893 and APX to upregulate co-stimulatory molecules on human B cells are similar, while the induction of co-receptor expression induced by MAB 271 appears to be lower (Figure 4a,b). While these differential activities are consistent within each donor and experiment, the mean fold-of-induction over four donors does not reach statistical significance due to the high donor-to-donor variations. 

### 3.6. Assessment of Pharmacodynamic Markers and Potential Side Effects of the CD40 Agonistic Antibody MAB 273 in Humanized Mice 

The preclinical and clinical experience with CD40-targeting therapeutic antibodies has shown proof of concept but has also demonstrated substantial limitations in current therapeutic molecules due to toxicities. Thromboembolic events and liver transaminase elevations have resulted in a maximum tolerated dose of only 0.2 mg/kg for CP-870,893 [25,26,27]. Therefore, an important question is whether the FcγR-independent mode of action translates into a relevant biological activity in vivo and whether this treatment is associated with toxic side effects. To assess this in the context of a human immune system in vivo, the most efficacious agonistic CD40-specific antibody, MAB 273, was tested in a humanized mouse model system. As a model system we made use of NOD/Scid/Il2rγ^−/−^/FcRγ^−/−^ mice, which lack functional mouse activating FcγRs and can be transplanted with human hematopoietic stem cells, allowing for the testing of human therapeutic antibody activity in the context of a human immune system [43,44,45,46]. For a comparison, the clinical antibody CP-870,893 was used. 

**Figure 5:**
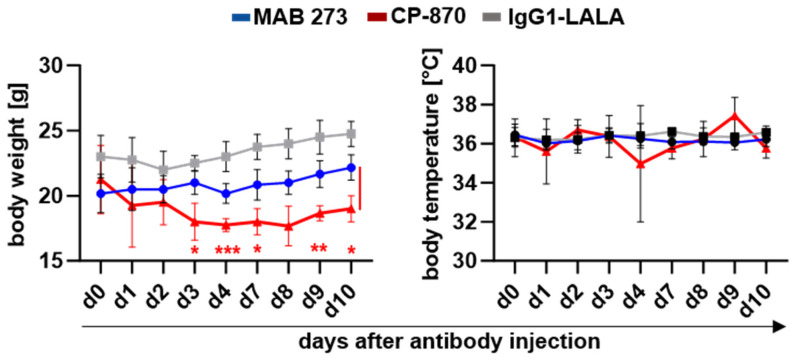
Changes in the body weight and body temperature of human-stem-cell-transplanted mice after anti-CD40 antibody injection. Antibodies were injected on day 0, and measurement was performed on different days after treatment. Depicted is the change in body weight (mean +/− SD) and body temperature (mean +/− SD) following the injection of the indicated antibodies and isotype controls. The injection of 4–6 mice per group with MAB 273 (*n* = 6; 10 mg/kg), IgG1-LALA (*n* = 6; 10 mg/kg) and CP-870,893 (*n* = 4; 3 mg/kg). Statistically significant differences between indicated groups were determined using two-way ANOVA. * *p* < 0.05, ** *p* < 0.01, *** *p* < 0.005.

As shown in Figure 5, a single injection of 3 mg/kg of CP-870,893 resulted a transient loss of body weight which became significant at day 3 after antibody injection. Across independent experiments, five out of thirteen mice that had been treated with 3 mg/kg CP-870,893 had to be sacrificed in response to treatment (Table S3). In contrast, treatment with 10 mg/kg MAB 273 was tolerated well, with no evident drop in body weight or temperature over the course of 10 days (Figure 5). Histological analyses of kidney and liver revealed no obvious signs of tissue damage or inflammation for either the MAB 273 or the CP-870,893 antibodies (Figure S4). However, in three CP-870,893-treated mice immunofluorescent staining analysis showed murine platelet and, in one mouse, murine macrophage infiltrations in the liver (Figure S4). There was a detectable increase in T-cell numbers in the livers of all MAB 273-treated mice but not in isotype control hIgG1-LALA- or CP-870,893-treated mice (Figure S4). Liver transaminase levels in serum were not elevated in any treatment group (Figure S5). 

**Figure 6:**
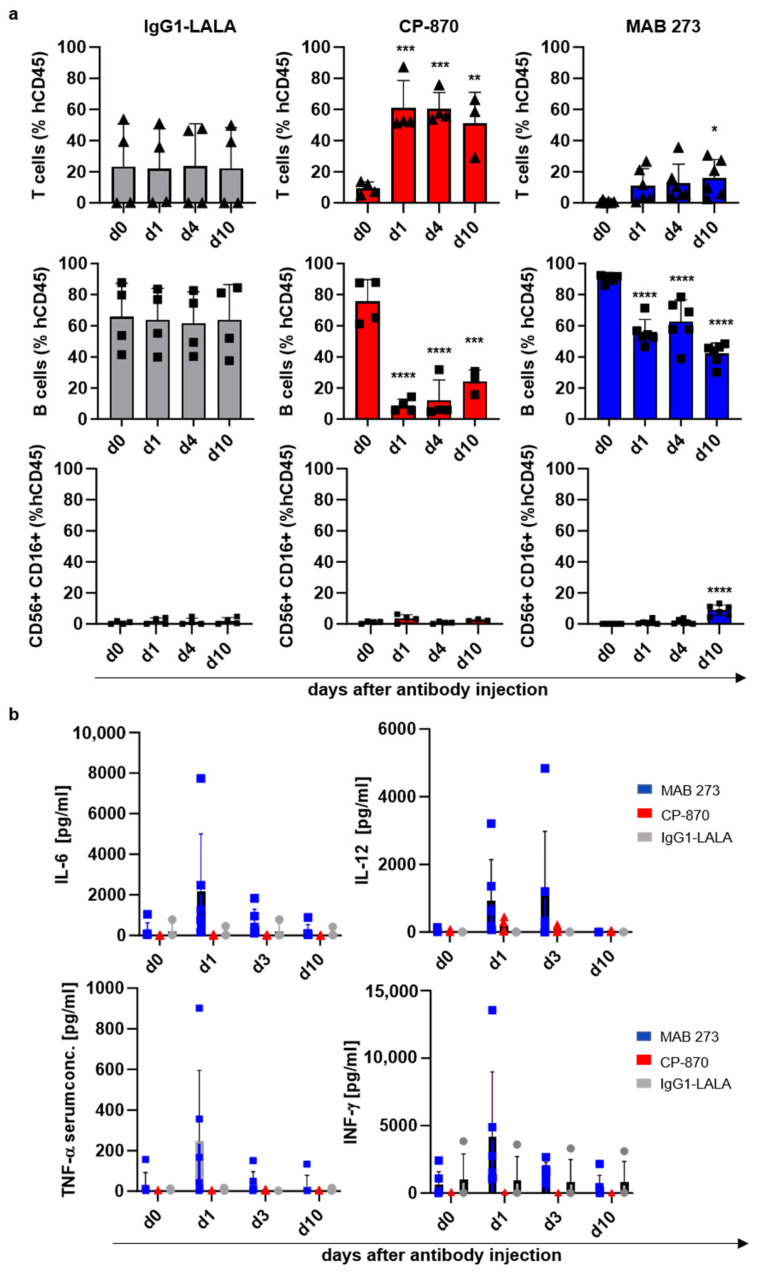
Effect of anti-CD40 antibody injection on human immune cell numbers and cytokine production in a human-stem-cell-transplanted mouse model. (**a**) Shown are changes in human immune cells in the peripheral blood of humanized mice injected with MAB 273, CP-870,893 or IgG1-LALA control antibodies as determined by flow cytometry. Bar graphs (mean + SD) with individual data points represent B-cell, T-cell and NK cell numbers as per cent of total human CD45+ cells in the peripheral blood of mice treated with the respective antibodies. Symbols depict individual mice. The statistical assessment of the data was performed with a one-way ANOVA test. * indicates a significant difference between the marked value and day 0. * *p* < 0.05, ** *p* < 0.01, *** *p* < 0.005, **** *p* < 0.001. (**b**) The bar graphs (mean + SD) depict the concentration of human IL-12p70, TNF-α, IL-6 and IFN-γ at the indicated time points after the injection of MAB 273, CP-870,893 and IgG1-LALA control antibodies. Symbols indicate individual mice. Cytokine concentrations in murine serum were determined using a LEGENDplex^TM^ Multi-Analyte Flow Assay. Values between groups did not reach statistical significance as determined by a one-way ANOVA.

We further tested how treatment with MAB 273 (10 mg/kg), CP-870,893 (3 mg/kg) or hIgG1-LALA (10 mg/kg) affected peripheral blood immune cell subsets and concentrations of cytokines in the plasma over the course of 10 days after a single antibody injection (Figure 6 and Figure S6). Confirming observations in other preclinical and clinical studies, we found an immediate disappearance of circulating B-cells in the blood in CP-870,893- and to a much lesser extent in MAB 273-treated mice (Figure 6a). The numbers of T-cells immediately increased in CP-870,893-treated mice, whereas in MAB 273-treated mice a slower increase in T cells was noted which became significant at day 10 after antibody injection (Figure 6a). Interestingly, we noted a small but highly significant increase in NK cells in the peripheral blood in MAB 273-treated mice 10 days after antibody injection (Figure 6a). Importantly, pro-inflammatory cytokines like IL12p70, IL-6, TNF-α and IFN-γ, promoting or being produced as a result of Th1 differentiation and proliferation of T- and NK-cells, were also increased in the serum of MAB 273- but not CP-870,893-treated mice, although at highly variable levels (Figure 6b). These in vivo results are in line with the activities of MAB 273 and CP-870,893 measured in vitro in moDCs and provide evidence that cells of the adaptive immune system are being activated in response to a single dose of MAB 273. 

In summary, MAB 273 treatment is tolerated well in humanized mice at the tested dose of 10 mg/kg, whereas treatment with 3 mg/kg CP-870,893 induced severe side effects, thus precluding treatment at higher doses.

## 4. Discussion

The triggering of CD40 receptor signaling on professional antigen-presenting cells has been shown to be a powerful way of supporting neo-antigen presentation to T-cells in a great number of preclinical studies, and clinical proof of concept has been demonstrated [2]. However, severe side effects in several clinical studies have limited using this immunomodulatory pathway to its full extent. For example, vascular thromboembolic events and liver toxicities have been observed in patients treated with the hIgG2 anti-CD40 antibody CP-870,893 and resulted in an MTD of only 0.2 mg/kg [27]. Apart from cancer immunotherapy, blocking IgG1 antibodies targeting CD40L for the therapy of autoimmune diseases has also been shown to cause cardiovascular thrombotic events in clinical studies [47]. Importantly, avoiding antibody-FcγR interactions by using either an anti-CD40L Fab’ or an aglycosylated IgG resulted in the prevention of such side effects in a preclinical study [23]. Recently, major progress has been made with respect to generating CD40-specific antibodies with a higher agonistic activity [2]. For example, the use of alternative isotypes, such as human IgG2, and the introduction of specific Fc-mutations with increased affinities to the inhibitory FcγRIIb have been shown to boost agonistic antibody activities in vivo [8,11,12,14,28,29]. However, at the same time, toxicities have increased, which has led to the development of intra-tumoral injection routes to prevent systemic toxicity and enhance the therapeutic window of opportunity [21,48]. 

In this study we show that by using a rabbit immunization approach, we successfully isolated and characterized an array of CD40-specific antibodies which efficiently activate CD40-mediated signaling. More importantly, the mode of action of these antibodies solely depends on the antibody paratope–epitope interaction and does not require FcγR binding neither in vitro nor in vivo. Notably, the most promising CD40-specific agonistic antibody candidates displayed a superior safety profile compared to the clinical CP-870,893 antibody. Interestingly, a recent study comparing a number of anti-CD40 antibodies binding to different sites on CD40 concluded that antibodies binding to extracellular domains comprising the CD40L-binding region are less potent agonists and that the N-terminal domain is the region of choice to elicit potent agonism [29]. Interestingly, the top four CD40-specific antibodies identified in our screen all interfered with CD40L binding to CD40 and bound to the receptor in a mutually exclusive fashion. In fact, we found several antibodies binding to that region, comprising functional as well as non-functional antibodies with respect to triggering IL-12 release by moDCs. Strikingly, the analysis of heavy- and light-chain CDR3 sequences of the top four antibodies shows that the clones are not just unrelated but very diverse and thus most probably bind in very different ways. Therefore, in addition to binding CD40 with high affinity, the paratope of the antibody seems to be the critical determinant for triggering canonical CD40 signaling, at least in our study. Whether the activity of our antibodies correlates with their affinity as described in a very recent study remains to be determined in future studies [30]. 

Of note, the most potent agonistic antibody identified in our study, MAB 273, displays a superior efficacy in vitro when compared with both the Fc-optimized CP-870,893-hIgG1-V11 and the CD40L-competing Fc-optimized hIgG1 APX antibodies. Importantly, this higher level of agonistic activity in vitro did not result in enhanced toxicity in vivo, whereas a single injection of 3 mg/kg CP-870,893 resulted in obvious toxicity. This is in line with the fact that in patients CP-870,893 is tolerated only at low doses, with dose limiting toxicities becoming apparent at 0.2 mg/kg. In particular, D-dimer elevations and thromboembolisms have been observed, indicating effects on platelet activation and coagulation. In our study it remains unclear what causes the fatal outcomes in CP-870,893-treated mice. Platelet infiltrates in liver tissue have been observed in some animals, but in all other analyzed mice liver and kidney histology looked normal. Coagulative necrosis in the liver, as it has been shown to occur in mice treated with anti-murine-CD40 antibody FGK-45, could not be observed [19,49]. However, it cannot be ruled out that microvascular occlusions in other organs occurred in those animals. It is evident, however, that the toxicity of the treatment is not related to systemic cytokine release. In fact, CP-870,893 does not induce cytokine levels in the blood plasma of treated mice at the tested dose. In contrast, MAB 273 treatment leads to increased blood plasma levels of pro-inflammatory cytokines such as IL-12, TNF-α, IL-6 and IFN-γ. This reflects the observations made in vitro, where IL-12 and TNF-α release by moDCs treated with MAB 273 was reproducibly demonstrated. These cytokines may also be responsible for the relative increases in T-cell and NK-cell counts which were observed subsequent to cytokine peak levels in MAB 273- but not CP-870,893-treated animals. The unique cytokine profile triggered by MAB 273 in humanized mice was also noted very recently in a study with non-human primates, strongly suggesting that the data may be translatable to the human system [50].

Another interesting observation is that CP-870,893 treatment strongly reduced B-cell numbers as it has been described in clinical studies. The disappearance of B-cells has been interpreted as a pharmacodynamic marker in clinical studies, but it is not clear whether this is due to redistribution or cell depletion. In the humanized in vivo model the effect of CD40-specific antibodies on B-cells does not correlate with blood cytokine levels. In both MAB 273- and CP-870,893-treated animals, peripheral B-cell counts are significantly reduced, but cytokines are induced only by MAB 273. Moreover, MAB 273 contains the LALA mutation and thus is not able to mediate an FcγR-dependent B cell depletion along the lines of a classical antibody-dependent cellular cytotoxicity (ADCC) reaction. This supports the idea that CP-870,893 binding to CD40 has qualitatively different functional consequences when compared with MAB 273 binding to CD40 in vivo. Currently, second-generation anti-CD40 antibodies carrying engineered Fc-parts for optimal FcγRIIb binding or incorporating IgG2 Fc-variants allowing optimal agonistic antibody activity are in the process of pre-clinical and clinical testing [2]. Whether the MAB 273 mediate T cell activation is sufficient to prime tumor-specific immune responses, allowing for the control of tumor growth in humanized mice, will be addressed in future studies. 

## 5. Conclusions

In summary, our study shows that potent CD40 agonistic antibodies not requiring Fc-effector functions can be isolated from rabbits, broadening the armamentarium of agonistic antibodies as stand-alone or as combination treatments with checkpoint blockade antibodies.

## Data Availability

The original contributions presented in the study are included in the article and Supplementary Materials, further inquiries can be directed to the corresponding authors.

## Supplementary Materials

The following supporting information can be downloaded at: https://www.mdpi.com/article/10.3390/antib13020031/s1, Figure S1: Induction of NF-kB signaling by 303 humanized hIgG1-LALA CD40 antibodies in HEK-Blue-CD40L™ gene reporter cells; Figure S2: CD40 antibody binding to HEK-Blue-CD40L™ cells in the presence of CD40L; Figure S3: Determination of size and aggregation state of MAB 271 and MAB 273 in solution; Figure S4: Immune-histology analysis of anti-CD40-antibody-treated human-stem-cell-transplanted mice; Figure S5: Analysis of ALT and AST plasma levels in humanized mice after anti-CD40 antibody treatment; Figure S6: Effect of anti-CD40 antibody injection on human cytokine production in humanized mice; Table S1: CDR sequence divergence of novel CD40 specific agonistic antibodies; Table S2: Affinity of different CD40 specific antibodies to CD40; Table S3: Pathogenicity of CD40 specific antibodies in human stem cell transplanted mice.
