# Supplementary material for: Fcγ-Receptor-Independent Controlled Activation of CD40 Canonical Signaling by Novel Therapeutic Antibodies for Cancer Therapy
Source: Antibodies (Basel). 2024 Apr 18;13(2):31. doi: 10.3390/antib13020031 (PMC11036229; doi:10.3390/antib13020031)
Supplement: Supplementary file 1 [file antibodies-13-00031-s001.zip › antibodies-2857541-supplementary.pdf]

Fcγ-receptor-independent controlled activation of CD40 canonical signaling by novel therapeutic antibodies for cancer therapy

Supplemental data:

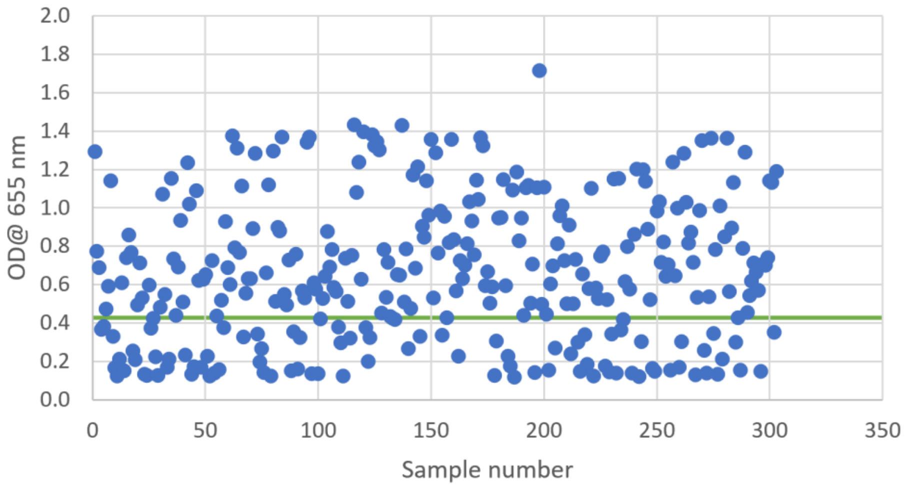

Figure S1: Induction of NF-κB signaling by 303 humanized hIgG1-LALA CD40 antibodies in HEK-Blue-CD40L™ gene reporter cells.

Antibodies were incubated for 6 hours at a concentration range of 80 to 10,000 ng/ml. Each dot indicates the maximum OD measured at 655 nm for each antibody. The green line indicates the threshold above which antibodies were classified as functional in this assay.

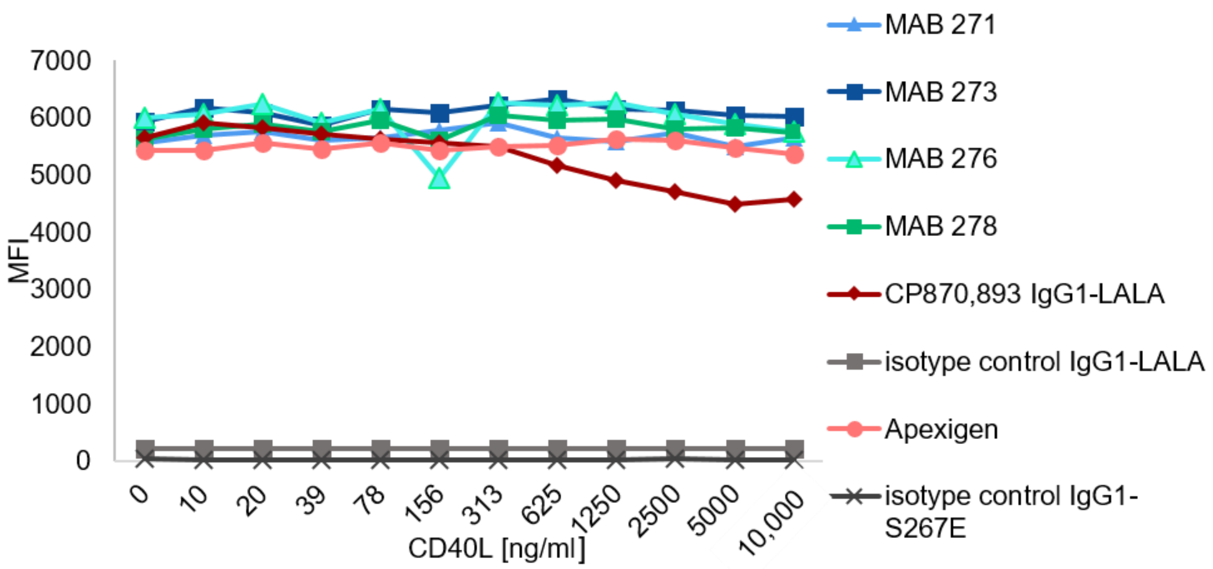

Figure S2: CD40 antibody binding to HEK-Blue-CD40L™ cells in the presence of CD40L.

Cells pre-incubated with CD40 antibodies or isotype controls were incubated with increasing concentrations of mouse-Fc-tagged CD40L. Antibody binding, shown as the mean fluorescence intensity (MFI), was detected using a fluorophore coupled anti-human IgG and analyzed by flow cytometry.

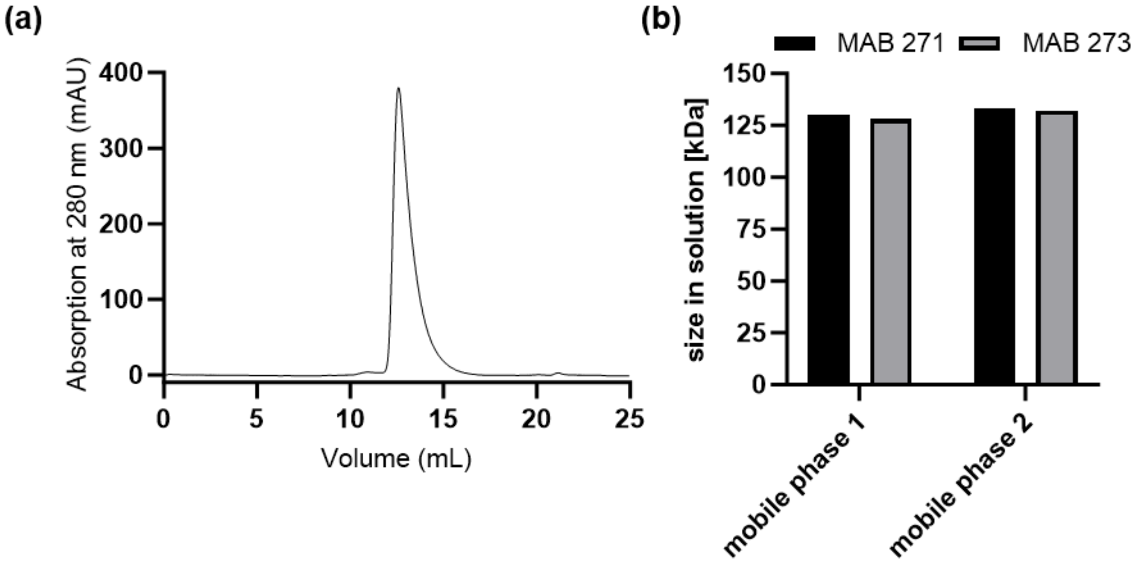

Figure S3: Determination of size and aggregation state of MAB 271 and MAB 273 in solution

(**a**) Depicted is a representative trace of a size exclusion chromatogram of MAB 273 depicting its monomeric form in solution. (**b**) Shown is a histogram of an analytical size exclusion chromatography depicting the size in solution of MAB 271 and MAB 273 under two different buffer conditions (mobile phase 1: 100 mM Phosphate, 200 mM Arginine, pH6,4; mobile phase 2: 100 mM Phosphate, 300 mM NaCl, pH6,4). Shown is one representative out of three independent experiments.

(a)

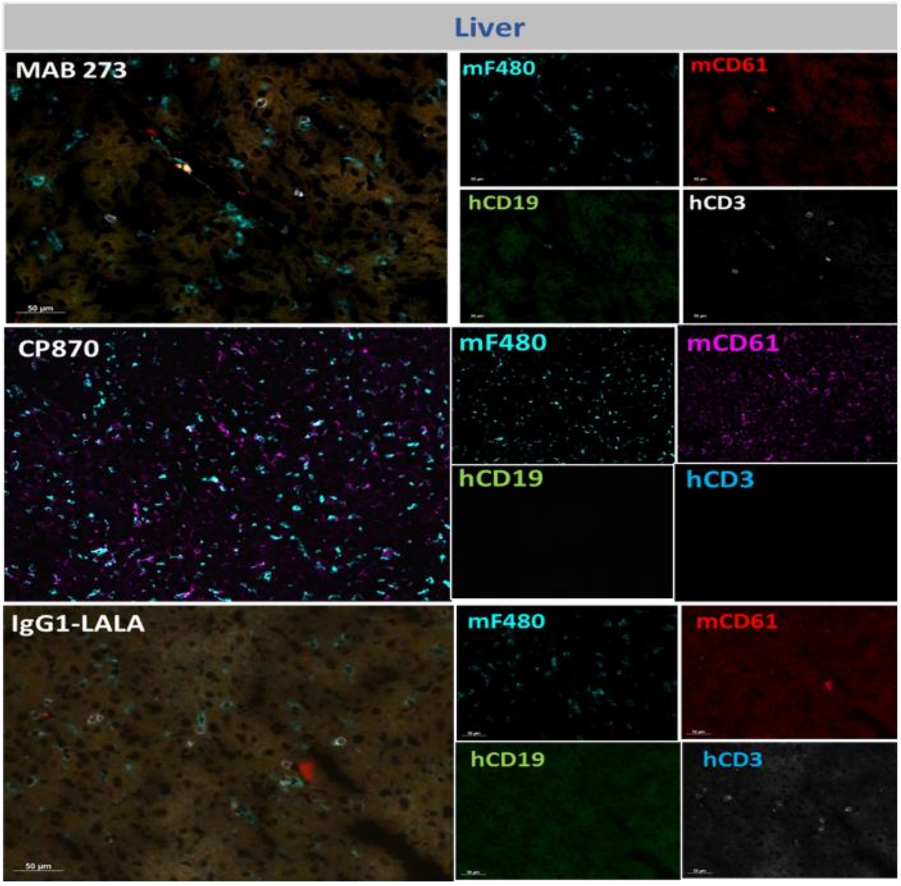
Liver
MAB 273
mF480
mCD61
hCD19
hCD3
CP870
mF480
mCD61
hCD19
hCD3
IgG1-LALA
mF480
mCD61
hCD19
hCD3

(b)

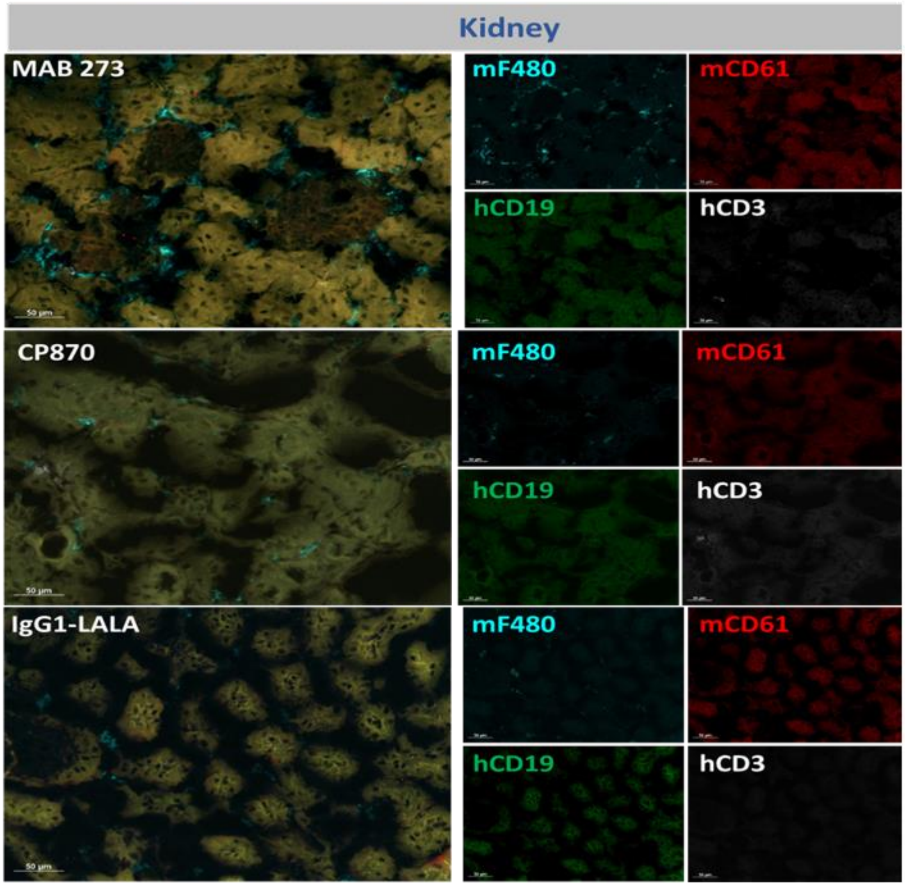
Kidney
MAB 273
mF480
mCD61
hCD19
hCD3
CP870
mF480
mCD61
hCD19
hCD3
IgG1-LALA
mF480
mCD61
hCD19
hCD3

Figure S4: Immune-histology analysis of anti-CD40-antibody-treated human-stem-cell-transplanted mice.

Shown is the analysis of liver (**a**) and kidney sections (**b**) of mice injected with MAB 273, CP-870,893 or hIgG1-LALA control antibodies. Sections were stained for mouse macrophage- (mF4/80), mouse platelet- (mCD61), human B cell- (hCD19) and human T cell- (hCD3) markers. White arrows indicate hCD3 positive T-cells detected in liver sections of MAB 273 treated mice.

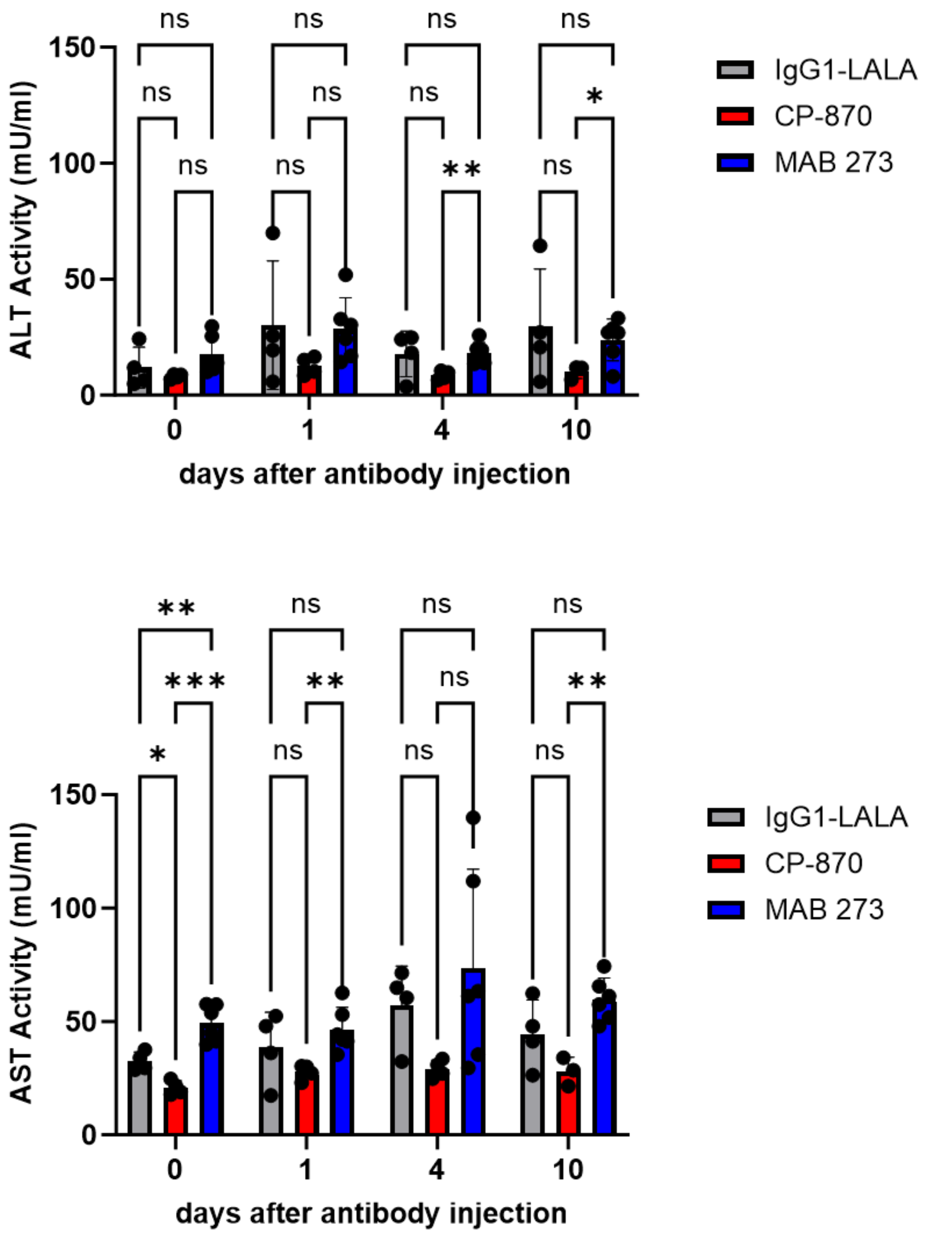

Figure S5: Analysis of ALT and AST plasma levels in humanized mice after anti-CD40 antibody treatment.

Shown are the concentrations of the liver transamidases ALT and AST determined in the serum of human stem cell transplanted mice injected with the respective antibodies at the indicated time-points after antibody injection. Each symbol indicates an individual mouse. Statistical assessment of the data was performed with a two-way ANOVA. Ns indicates no statistically significant different value between indicated groups. *p<0.05, **p<0.01, ***p<0.005, ns = not significant.

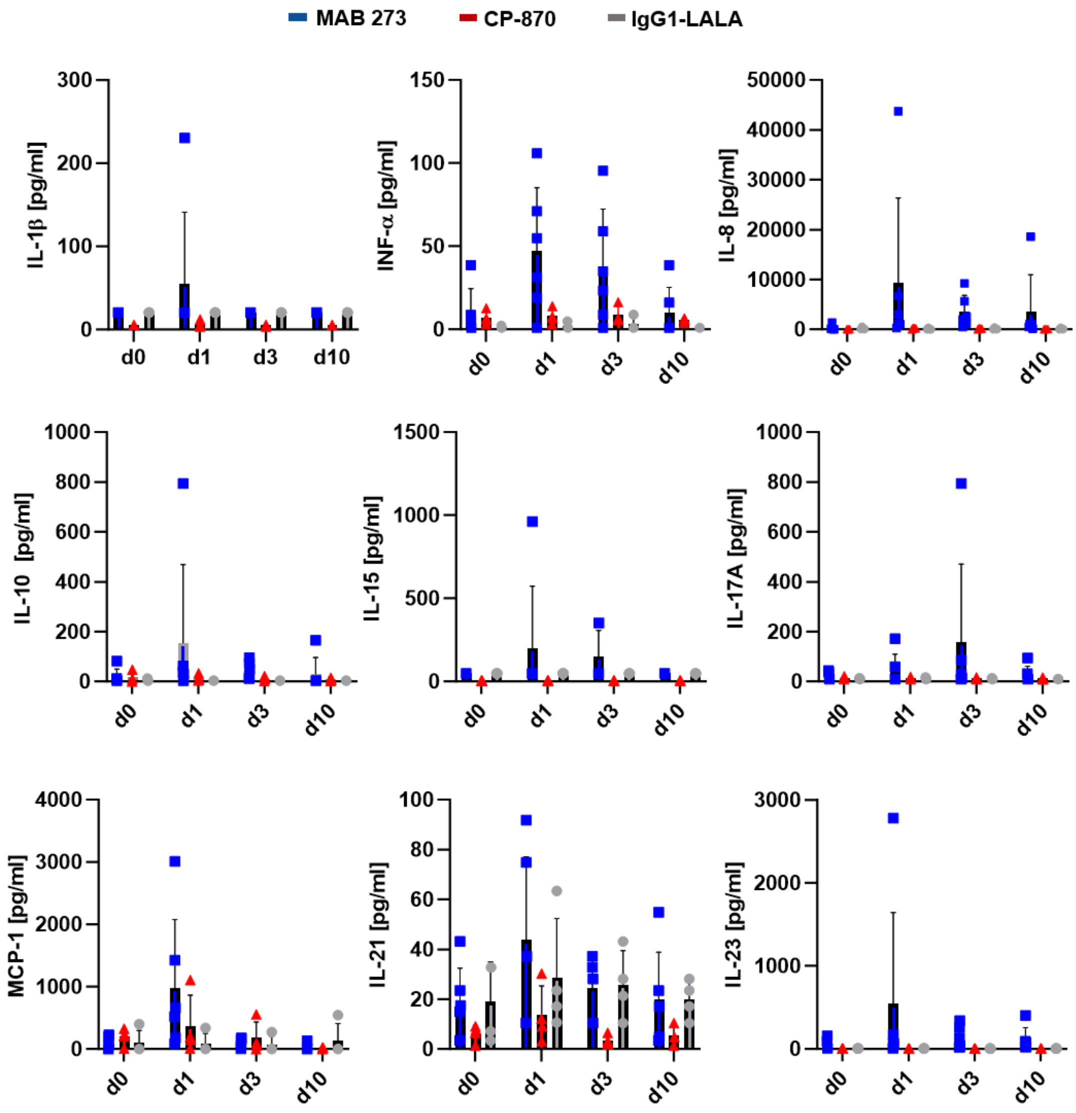

Figure S6: Effect of anti-CD40 antibody injection on human cytokine production in humanized mice.

The graphs depict the concentration of the indicated human cytokines at the indicated time points after injection of MAB 273, CP-870,893 and IgG1-LALA control antibodies. Symbols indicate individual mice. Cytokine concentrations in murine serum were determined using a LEGENDplex^{TM} Multi-Analyte Flow Assay. Statistical assessment of the data was performed using a two-way ANOVA. The data between groups did not reach statistical significance.

Table S1: CDR sequence divergence of novel CD40 specific agonistic antibodies.

Heavy chain CDRs				Light chain CDRs			
	**MAB 273**	**MAB 276**	**MAB 278**		**MAB 273**	**MAB 276**	**MAB 278**
**MAB 271**	20%	37%	56%	**MAB 271**	48%	53%	48%
**MAB 273**	-	30%	50%	**MAB 273**	-	56%	52%
**MAB 276**	-	-	58%	**MAB 276**	-	-	53%

Differences of amino acids at defined positions are indicated as % difference for the heavy and light chain CDR sequences (HC/LC).

Table S2: Affinity of different CD40 specific antibodies to CD40

Antibody	k_a ($M^{-1}s^{-1}$)	k_d (s^{-1})	K_D (nM)
MAB 273	$(9.74 \pm 0.37) \times 10^{5}$	$(1.18 \pm 0.05) \times 10^{-3}$	**1.2 ± 0.0**
MAB 271	$(7.25 \pm 0.53) \times 10^{5}$	$(1.13 \pm 0.01) \times 10^{-2}$	**15.7 ± 1.1**
CP-870	$(1.51 \pm 0.08) \times 10^{5}$	$(1.34 \pm 0.13) \times 10^{-3}$	**8.9 ± 1.4**
APX	$(6.89 \pm 0.06) \times 10^{5}$	$(7.29 \pm 0.13) \times 10^{-3}$	**10.6 ± 0.1**

Table S3: Pathogenicity of CD40 specific antibodies in human stem cell transplanted mice

Treatment	Total number of mice	Number of euthanized mice
**MAB 273**	**9**	**0**
**CP-870,893**	**13**	**5**
**IgG1-LALA**	**8**	**0**
